# Genetic characterization of *Escherichia coli* and *Klebsiella* spp. from humans and poultry in Nigeria

**DOI:** 10.1099/acmi.0.000509.v4

**Published:** 2023-07-12

**Authors:** Christiana Jesumirhewe, Adriana Cabal-Rosel, Franz Allerberger, Burkhard Springer, Werner Ruppitsch

**Affiliations:** ^1^​ Department of Pharmaceutical Microbiology, College of Pharmacy, Igbinedion University, Okada, Edo state, Nigeria; ^2^​ Institute of Medical Microbiology and Hygiene, Austrian Agency for Health and Food Safety, Vienna, Austria

**Keywords:** antibiotic resistance, *Escherichia coli*, human, *Klebsiella* spp., poultry, whole genome sequencing

## Abstract

The emergence of antibiotic resistance in livestock, especially food-producing animals, is of major public health importance as a result of the possibility of these bacteria entering the food chain. In this study, the genetic characteristics of antibiotic-resistant *Escherichia coli* and *Klebsiella* spp. isolates from humans and poultry in Edo state, Nigeria, were investigated. In April 2017, 45 *Klebsiella* spp. and 46 *E. coli* isolates were obtained from urine, clinical wounds, nasal and chicken faecal samples. Isolates were recovered and identified as previously described. Species identification was achieved by matrix-assisted laser desorption ionization-time of flight (MALDI-TOF) mass spectrometry and ribosomal multilocus sequence typing. Antimicrobial susceptibility testing was carried out using the Kirby–Bauer method for 12 antibiotics. A double disc synergy test was used to screen for extended-spectrum beta-lactamse (ESBL) production. Whole genome sequencing was performed for strain characterization of the isolates. Thirteen *Klebsiella* spp. isolates yielded positive results by the ESBL phenotypic test and harboured ESBL genes. Of the 46 *E. coli* isolates, 21 human and 13 poultry isolates were resistant to at least one of the tested antibiotics. Four human *E. coli* isolates harboured ESBL genes and revealed positive results when applying ESBL double disc synergy tests. ESBL genes in the *Klebsiella* spp. and *E. coli* isolates include *bla*_CTX-M-15_ and *bla*_SHV-28_. Whole genome-based core gene multilocus sequence typing of the *Klebsiella* spp. and *E. coli* isolates revealed a close relatedness among the isolates. An integrated ‘One Health’ surveillance system is required to monitor transmission of antimicrobial resistance in Nigeria.

## Data Summary

This Whole Genome Shotgun project has been deposited at the DDBJ/EMBL/GenBank under accession JAMZSQ000000000-JAMZTV000000000. The version described in this paper is version JAMZSQ010000000–JAMZTV010000000

## Introduction

Antimicrobial resistance (AMR) is an increasing worldwide health challenge that is regarded as a ‘One Health’ concern affecting human health, animal health and the environment. Antimicrobials are usually used for prophylaxis and therapeutic purposes in humans and animals. The use of antimicrobials especially in livestock for disease control and promotion of growth promotes the selection and propagation of resistant strains. These resistant strains can be transferred from the animals to humans and the environment [1]. The main mechanisms of antibiotic resistance include limiting uptake of a drug, modification of a drug target, and inactivation and active efflux of a drug [2]. These mechanisms may be intrinsically present or be acquired by mutations or transfer of resistance determinants. AMR is most commonly associated with elements that are extra-chromosomally located, including different types of mobile genetic elements such as plasmids, transposons and integrons acquired from other bacteria [3]. A large number of antimicrobials are used in poultry farming in most countries including Nigeria [4, 5]. Nigeria’s poultry production has grown steadily and the unrestricted use of antimicrobials is common. Resistance in *Escherichia coli *, *Staphylococcus* spp., *Salmonella* spp.*, Bacillus* spp. and *Klebsiella* spp. has been previously reported in poultry farms [6, 7]. Previous Nigerian studies have reported resistance in isolates from poultry to some important antibiotics such as tetracycline, ciprofloxacin, sulphonamides, gentamicin and trimethoprim [4, 8].

AMR in species of the family *Enterobacteriaceae,* especially *Escherichia coli* and *Klebsiella pneumoniae,* has spread globally causing infections that are difficult to treat [9, 10]. Only a few Nigerian studies have characterized antibiotic resistance genes in poultry isolates [4, 11, 12]. Genetic characterization of AMR genes may give further insight into transmission mechanisms representing a key prerequisite for control of the spread of AMR [13]. The present study aims to characterize AMR *E. coli* and *Klebsiella* spp. isolates recovered from humans and poultry in Edo state Nigeria using whole genome sequencing.

## Methods

### Study site and sample collection

In April 2017, urine samples (130) and nasal samples (50) were obtained from healthy students of the College of Pharmacy, Igbinedion University Okada, Edo, Nigeria. The sample sizes were randomly determined. Blinding of participants was not required for the study. No inclusion or exclusion criteria were used in obtaining the samples. The samples were transported to the Department of Pharmaceutical Microbiology Laboratory immediately after collection. Isolates from clinical wounds (70) were collected from the medical microbiology laboratory of the University of Benin teaching hospital, Edo state, Nigeria. Isolates obtained were from samples of both inpatients and outpatients of the hospital. During the same period, four poultry farms situated in Okada and Benin city Edo state, Nigeria, were visited. All farms were visited once and 100 chicken faecal samples were collected and transported to the Department of Pharmaceutical Microbiology Laboratory immediately after collection. Identification of isolates was achieved using standard microbiological techniques [14]. *E. coli* and *Klebsiella* spp. were isolated and identified by inoculating samples on MacConkey agar plates (Oxoid) and incubating for 24 h at 37 °C. Distinct colonies obtained from the agar plates were subcultured to obtain pure colonies. Species identification was achieved by using matrix-assisted laser desorption ionization-time of flight (MALDI-TOF) MS (Bruker Daltonik) analysis.

### Ethical considerations

Research ethics approval was not required in the hospital where isolates were obtained for the study. Only pre-identified isolates were obtained from the microbiology laboratories and used in the study. There was no contact with patients and the samples from the hospital. The institution did not require informed consent. Data regarding the isolates were obtained from clinical records and analysed anonymously. Concerning the samples obtained from the healthy students, approval from the Igbinedion university ethical committee was duly obtained for this study. The ethical document iuo/ethics/22/009 was initiated for the study. Samples were obtained from both male and female students. Data on the age and weight of the students were not obtained for the study. All participants were informed about the study’s purpose and procedures. Informed consent was obtained from the students before samples were collected.

### ESBL detection and antibiotic susceptibility testing

The Kirby–Bauer susceptibility testing technique [15] was carried out and results were interpreted using European Committee on Antimicrobial Susceptibility Testing criteria [16]. The isolates were tested with 12 antibiotics: meropenem, ertapenem, cefotaxime, amoxicillin/clavulanic acid, cefoxitin, cefepime, tigecycline, ciprofloxacin, amikacin, ampicillin, cefuroxime and gentamicin (Oxoid). The isolates were tested for the production of extended-spectrum beta lactamses (ESBLs) using the double disc synergy test [17]. Confirmation of ESBL production was carried out as previously described in the CLSI guidelines [18]. An ESBL producer was defined by an increase of ≥5 mm in the inhibition zone diameter for cefotaxime or ceftazidime combined with clavulanic acid against the inhibition zone diameter of either cefotaxime or ceftazidime without clavulanic acid [18]. Based on a random selection criterion, some antibiotic-resistant and ESBL-positive isolates were selected for characterization by whole genome sequencing (WGS).

### Whole genome sequencing

WGS was carried out as previously described [1] for 16 resistant/multidrug-resistant *E. coli* and *Klebsiella* spp. isolates respectively based on a random selection criterion. Briefly, genomic DNA (gDNA) was extracted from the isolates using MagAttract HMW DNA extraction kit (Qiagen). Fragment libraries of the bacterial genomes were prepared using the Illumina Nextera XT DNA library preparation kit (Illumina). Library preparation of the genomes was followed by paired-end sequencing using a read length of 2×300 bp on an Illumina Miseq instrument (Miseq v3.0; Illumina). Assembly of raw reads (FASTQ files) was carried out using Velvet version 1.1.04 [19]. Ribosomal multilocus sequence typing (rMLST; https://pubmlst.org/species-id) was used to confirm the identities of the isolates. The ResFinder 2.1 web server tool (http://www.genomicepidemiology.org) [20] and the Comprehensive Antibiotic Resistance Database-Resistance Gene Identifier (CARD-RGI) [21] were used to identify plasmids and AMR genes in assembled genomes. The VirulenceFinder 2.0 web server tool (http://www.genomicepidemiology.org) [22] and the VFDB [23] were used to identify virulence factors in assembled genomes of *E. coli* and *Klebsiella* spp. isolates respectively. O- and H-types in the *E. coli* isolates were identified using SerotypeFinder 2.0 (http://www.genomicepidemiology.org) [24]. Mobile genetic elements and their relationship to AMR genes and virulence factors in assembled genomes were predicted using the MobileElementFinder (v1.0.2) (http://www.genomicepidemiology.org) [25]. For phylogenetic analysis, the MLST (multi-locus sequence typing) [26] and the *K. pneumoniae* core genome MLST (cgMLST) (https://www.cgmlst.org/ncs/schema/2187931/) were determined using SeqSphere+software v6.0.0 (Ridom). For strain comparison of *Klebsiella* spp.*,* minimum spanning trees (MSTs) were calculated based on the cgMLST scheme comprising 2358 core genes using SeqSphere+ software v6.0.0 (Ridom). A CT distance of 15 alleles was used to identify related isolates. The MLST and cgMLST scheme comprising 2513 core genes was used for *E. coli* strain comparison using SeqSphere+ version 6.0.0 (Ridom). Related *E.coli* isolates were identified with a CT distance of 10 alleles. ‘Good core genome targets’ was defined based on the criteria previously described in detail in Ruppitsch *et al*. [27]. Plasmids on the draft genomes of the resistant *E. coli* and *Klebsiella* spp. isolates were detected and classified using the PlasmidFinder 1.3 webtool (https://cge.food.dtu.dk/services/PlasmidFinder/) [28]. Replication of experiments was not required in the study.

## Results

### Antibiogram and phenotypic resistance pattern of the isolates

**Fig. 1.**
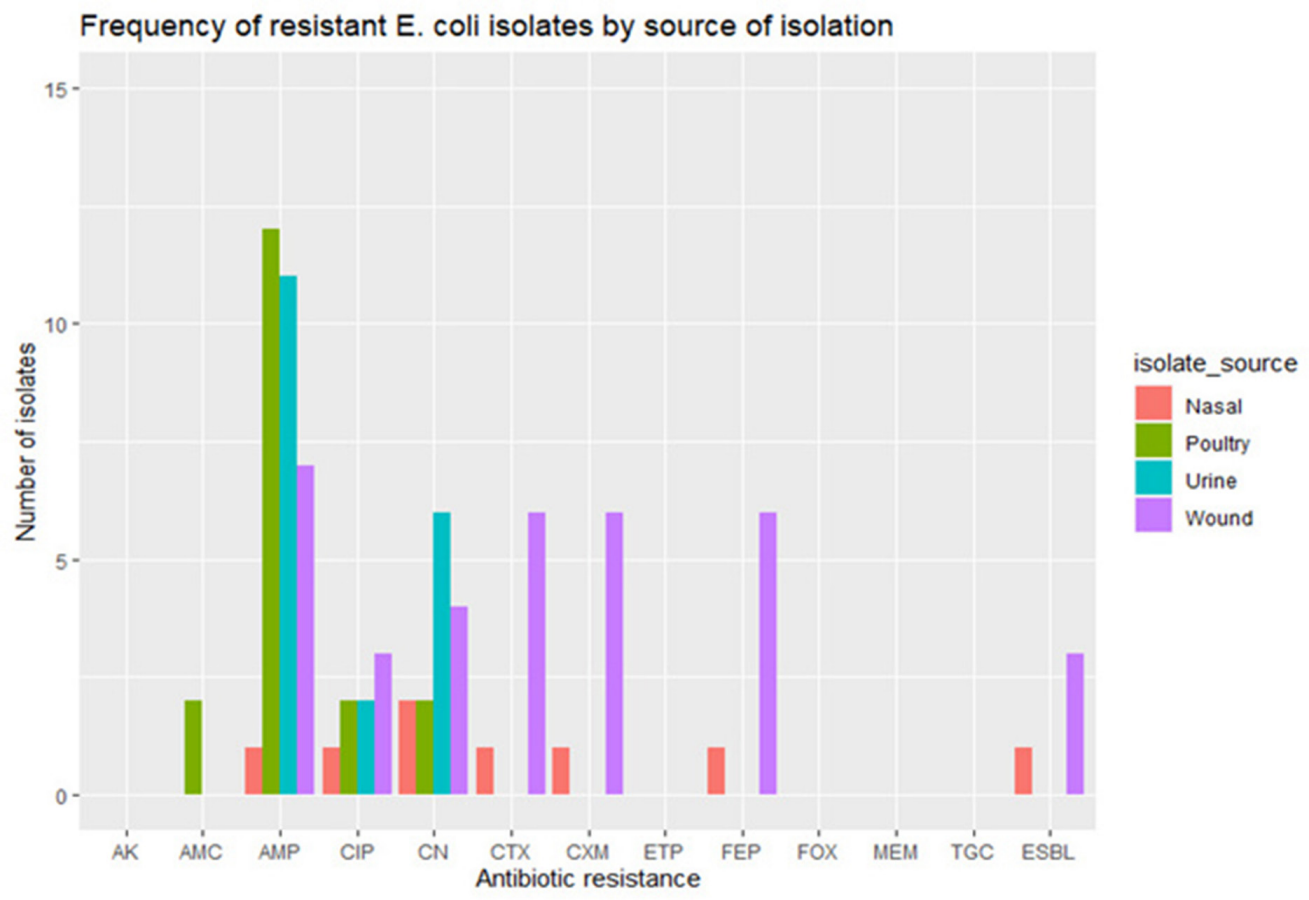
Frequency of the resistant *E. coli* isolates.

**Fig. 2.**
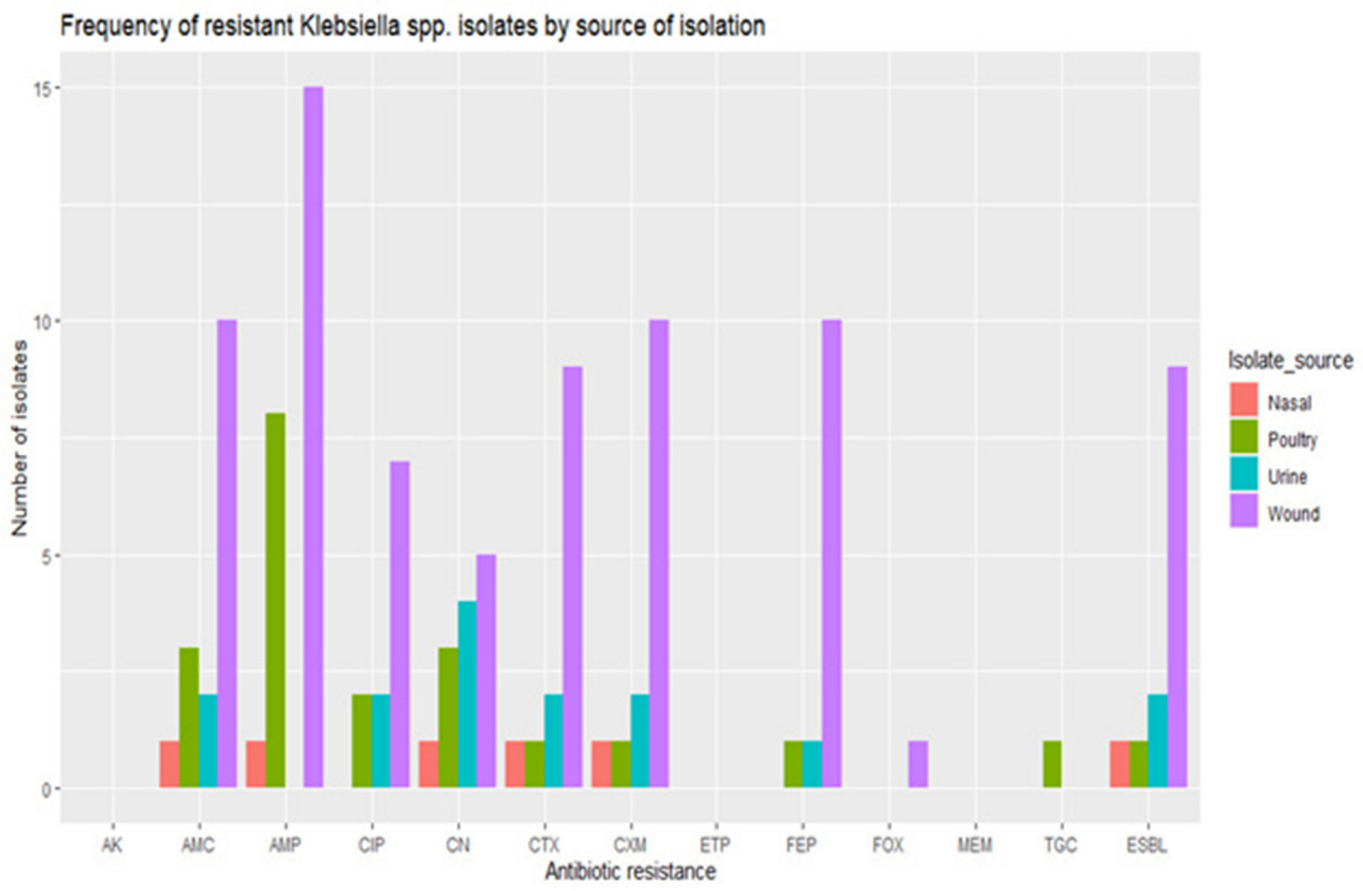
Frequency of the resistant *Klebsiella* spp. isolates.

Of the 46 *E. coli* isolates, 21 human (two nasal, 12 urine, seven wound) and 13 poultry isolates were antibiotic resistant in the Kirby–Bauer susceptibility testing technique. Four human (three wound, one nasal) *E. coli* isolates showed positive results in the ESBL double disc synergy tests. The ESBL *E. coli* isolates were all resistant to cefepime, cefuroxime, cefotaxime and gentamicin. Only one ESBL *E. coli* was resistant to ciprofloxacin. Twelve human (nine wound, two urine, one nasal) and one poultry *Klebsiella* spp. isolates showed positive results when applying ESBL double disc synergy tests. All ESBL *Klebsiella* spp. isolates were resistant to amoxicillin clavulanic acid, cefuroxime and cefotaxime. Eleven ESBL *Klebsiella* spp. isolates were cefepime-resistant. Ten and seven ESBL *Klebsiella* spp. isolates were also resistant to ciprofloxacin and gentamicin respectively (Figs 1 and 2).

### AMR genes of the recovered resistant isolates

ESBL genes detected among the isolates included *bla*_CTX-M-15_ and *bla*_SHV-28_. Multiple resistance determinants were detected on the draft genome sequence of the 12 human and four poultry *E. coli* isolates analysed (Table S1, available in the online version of this article). The resistance determinants included β-lactamase genes, aminoglycoside modifying enzymes, fosfomycin resistance determinants, *qnr* genes, sulphonamide resistance genes, tetracycline resistance genes, trimethoprim resistance genes and phenicol resistance genes. Other resistance determinants, which included efflux regulatory systems modulating antibiotic efflux, antibiotic target alteration and elfamycin resistance, were also detected in the antibiotic-resistant *E. coli* isolates. Eleven plasmid incompatibility groups were identified among the 16 resistant isolates with IncF family types being predominant. WGS also confirmed the presence of other β-lactamase genes, aminoglycoside-modifying enzymes, fosfomycin resistance determinants, *qnr* genes and plasmid encoded efflux pump, sulphonamide resistance genes, trimethoprim resistance genes, phenicol resistance genes among the resistant *Klebsiella* spp. isolates (Table S1). Thirteen plasmid incompatibility groups were identified among the resistant *Klebsiella* spp. isolates with IncF family types also being predominant.

### Virulence factors of the recovered resistant isolates

Known virulence determinants involved in adherence, biofilm formation, capsule synthesis regulation, immune evasion, secretion system, serum resistance, siderophore expression (enterobactin, yersiniabactin, aerobactin and salmochelin) and efflux pump expression were detected among the *Klebsiella* spp. isolates (Table S1). The chromosomal gene *fim*, *mrk* for adherence and biofilm formation, the immune evasion factor *cps*A which determines the polysaccharide capsule (K antigen) type, capsule synthesis regulation (*rcs*) serum resistance factor (*Omp*A) which determines the ‘O-antigen’ lipopolysaccharide serotype and efflux pump expression (*acrAB*) were identified among the *Klebsiella* spp. isolates. Other factors identified among the *Klebsiella* spp. isolates include factors the determine siderophores (iron carriers) [aerobactin (*iut*A), salmochelin (*iro*E, *iro*N) and enterobactin (*ent, fep*)] and type VI secretion system loci genes (Table S1).

For the *E. coli* isolates, all 16 isolates were predicted to be of four different serogroup O types by SerotypeFinder 2.0, and seven different H types were detected. Among the isolates, three (19 %) were identified as serotype O1:H25. Serotypes O8:H9, O7:H4, O8:H17 and O16:H5 were detected in four different *E. coli* isolates. Nine (56 %) *E. coli* isolates had only the serogroup H type by SerotypeFinder 2.0, which was predominantly H9 in six (38 %) *E. coli* isolates. MLST analysis showed that the six *E. coli* isolates with serogroup H9 belonged to sequence type (ST)155, three isolates with serogroup O1:H25 belonged to ST1722 and two isolates with ST10 were predicted to have serogroup H27. Six different *Fim*H types (23, 24, 32, 41, 54, 153) were identified among all the *E. coli* isolates except one in which it was not detected. Different virulence factors belonging to the four main virulence classes of *E. coli* pathotypes, namely colonization, fitness, toxins, and effectors, were identified among the *E. coli* isolates (Table S1). Some virulence genes were commonly detected among the *E. coli* isolates, which include *hly*E, *fim*H, gad, *nlpl*, *csg*A and *ter*C (Table S1). Other virulence factors detected include *chu*A, coding for an outer membrane haemin receptor, *yfc*V (a fimbriae adhesin), *iha, air, tia* and *lpf*A adhesins, *fyu*A expressing a siderophore receptor, *sen*B which codes for an enterotoxin, *eil*A, a *Salmonella* HilA homologue, *hra*, a heat-resistant agglutinin gene, *irp*2, encoding a non-ribosomal peptide synthetase, *pap*C, encoding outer membrane usher P fimbriae, *iss,* an increased serum survival protein, *cnf*1, a cytotoxic necrotizing factor, *kps*E a capsule polysaccharide export inner-membrane protein, and *tra*T, an outer membrane complement resistance protein (Table S1).

### Characterization of mobile genetic elements

MobileElementFinder (http://www.genomicepidemiology.org) predicted five types of mobile genetic elements (MGEs) among the resistant isolates, which include miniature inverted repeats, insertion sequences (ISs), an integrative conjugative element, unit transposons and composite transposons of which the majority were ISs. Some of the predicted ISs among the resistant isolates include ISEcl1, ISEc9, ISEc31, IS6100, IS26, ISKpn19 and ISKpn26. No integrative conjugative element ICEEoED1a-1 or miniature inverted repeat MITEEc1 was detected in *E. coli* and *Klebsiella* spp. isolates respectively. The majority of the resistant isolates had mobile elements in association with AMR and virulence genes. Seven *E. coli* isolates had mobile elements with no association with AMR or virulence genes (Table S1).

### Multilocus sequencing of the *Klebsiella* spp. and *E. coli* isolates

**Fig. 3.**
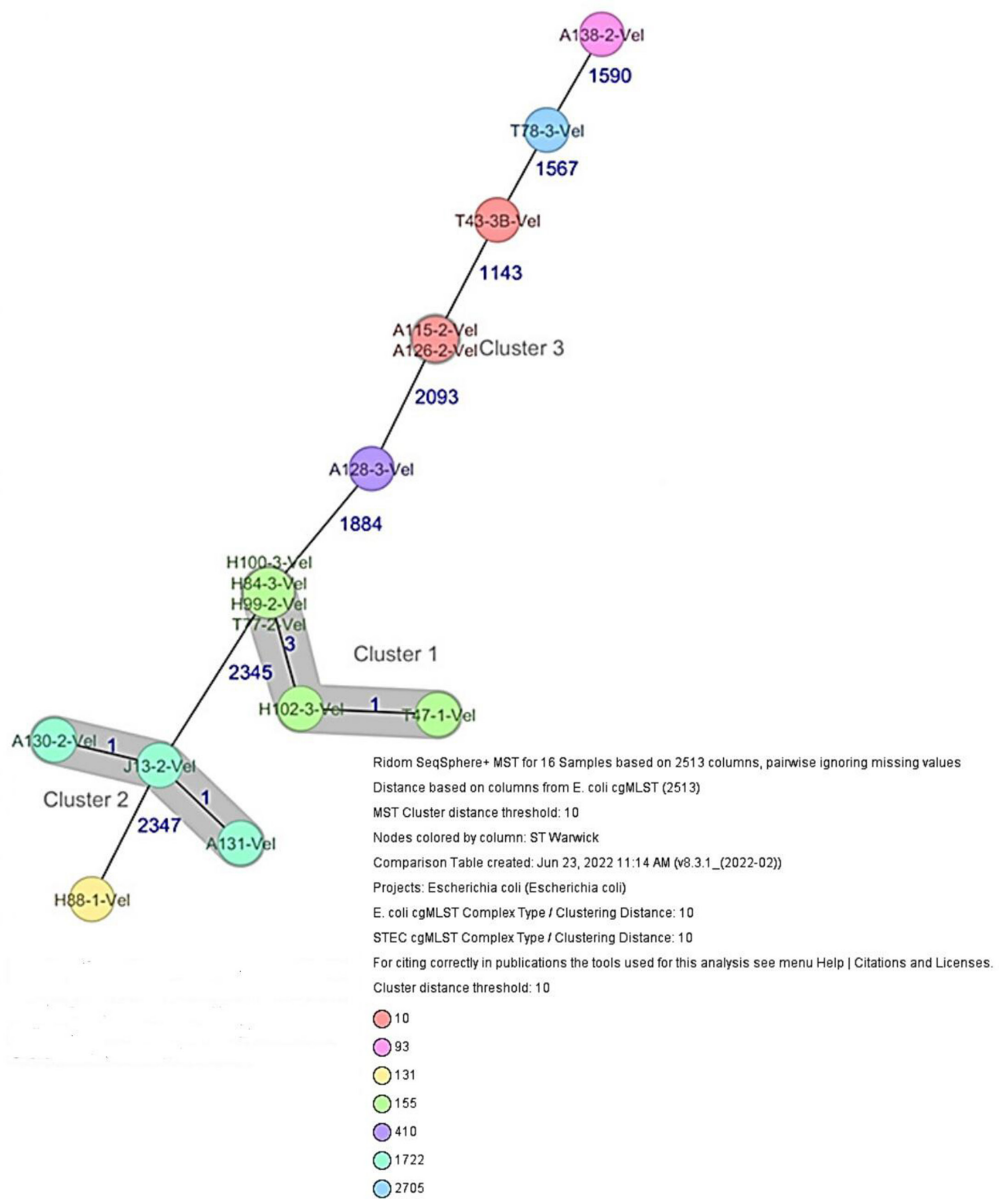
Minimum spanning tree (MST) based on cgMLST analysis of 16 *E. coli* isolates derived from healthy poultry, healhy human and clinical sources.

**Fig. 4.**
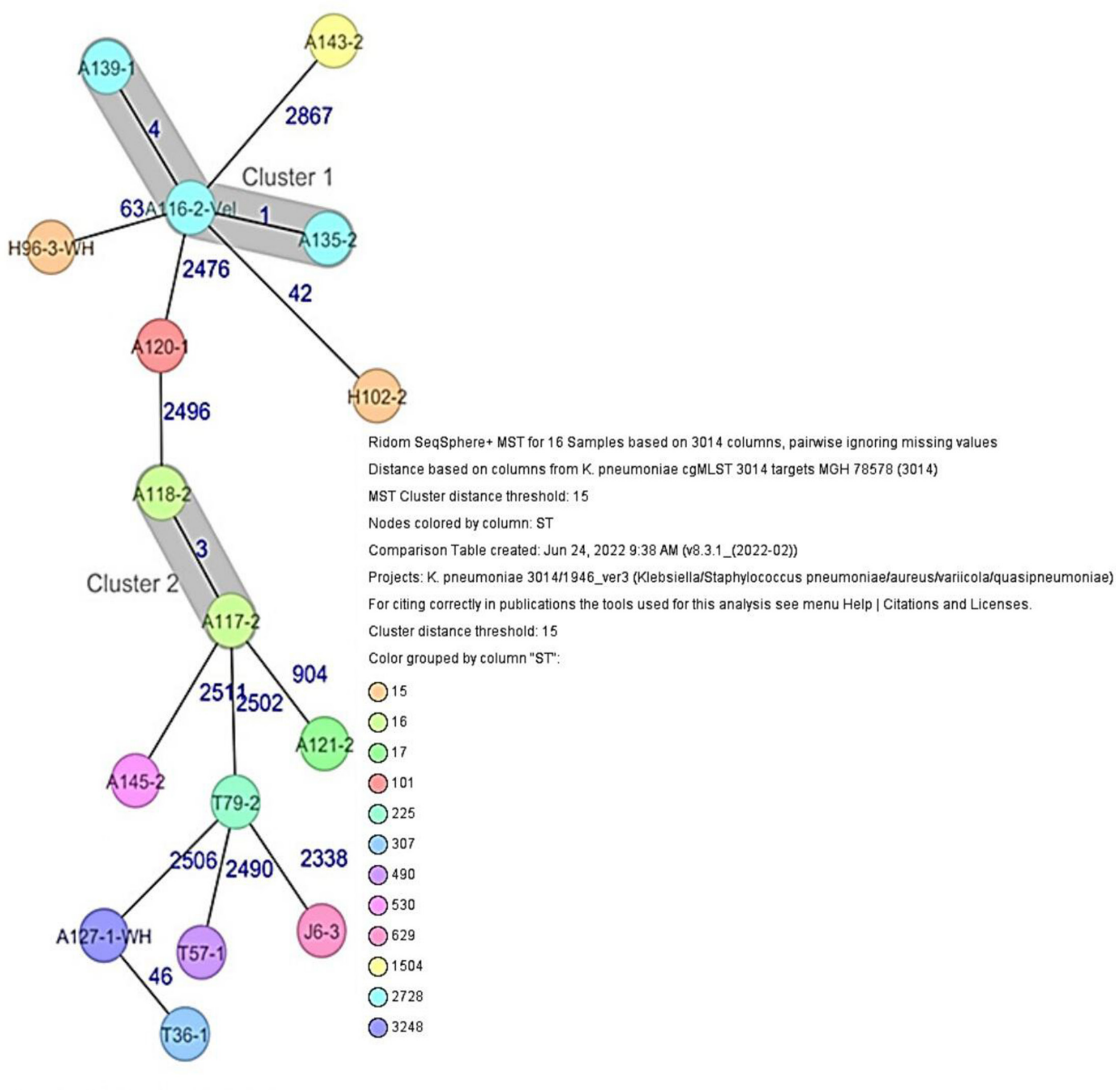
Minimum spanning tree (MST) based on cgMLST analysis of 16 *Klebsiella* spp. isolates derived from healthy poultry, healthy human and clincal sources.

The 16 sequenced antibiotic-resistant *E. coli* isolates from humans and poultry revealed seven different STs. Three different sequence types, ST10 (*n*=1), ST155 (*n*=2) and ST2705 (*n*=1), were detected among the poultry *E. coli* isolates. Among the human *E. coli* isolates (*n*=12), six different STs were identified, with ST155 being most prevalent (*n*=4). Other STs detected among the human *E. coli* isolates included ST10 (*n*=2), ST410 (*n*=1), ST1722 (*n*=3), ST93 (*n*=1) and ST131 (*n*=1) (Table 1). The overlap of STs between the two different sources (human and poultry) involved one sequence type (ST155) comprising two poultry and four human *E. coli* isolates (Fig. 3). The 16 resistant *Klebsiella* spp. isolates isolated from human specimens and poultry had 12 different STs. The three poultry *Klebsiella* spp. isolates had different sequence types, ST225, ST307 and ST490. Nine different STs were identified among the human *Klebsiella* spp. isolates with ST2728 being most prevalent (*n*=3). Other STs detected among the human *Klebsiella* spp. isolates include ST629 (*n*=1), ST15 (*n*=2), ST16 (*n*=2), ST101 (*n*=1), ST17 (*n*=1), ST1504 (*n*=1), ST530 (*n*=1) and ST3248 (*n*=1) (Table 1, Fig. 4).

**Table 1.** Sequence type and plasmid type of the isolates

Sample ID	Organism	Sequence type (ST)	Plasmid type
A115-2	* Escherichia coli *	10	IncFII; IncQ1
A126-2	* Escherichia coli *	10	IncFII; IncQ2
A128-3	* Escherichia coli *	410	IncFII - 3 x; IncFIA; IncFIB; IncN
A130-2	* Escherichia coli *	1722	IncFIA; IncFIB
A131	* Escherichia coli *	1722	IncFIA; IncFIB
A138-2	* Escherichia coli *	93	IncY
H84-3	* Escherichia coli *	155	IncFII
H88-1	* Escherichia coli *	131	IncFII; IncFIA; IncFIB; IncI1; Col156
H99-2	* Escherichia coli *	155	IncFII; IncQ1; p0111
H100-3	* Escherichia coli *	155	p0111; IncQ1; IncFII
H102-3	* Escherichia coli *	155	p0111; IncQ1
J13-2	* Escherichia coli *	1722	IncFIA; IncFIB
T43-3B	* Escherichia coli *	10	IncFII - 2 x
T47-1	* Escherichia coli *	155	IncQ1; p0111
T77-2	* Escherichia coli *	155	IncFII; IncQ1; p0111
T78-3	* Escherichia coli *	2705	p0111; IncXI1
J6-3	* Klebsiella pneumoniae *	629	IncFIB(K); IncFIB; IncHI1B
T36-1	* Klebsiella pneumoniae *	307	IncFIA(HI1); IncFIB(K); Inc R; IncFII(K)
T57-1	* Klebsiella pneumoniae *	490	Col440I; IncFIB(K); repA
T79-2	* Klebsiella pneumoniae *	225	IncFIB(K); IncFII; IncHI1B; IncN
H96-3	* Klebsiella pneumoniae *	15	Col440I; IncFIB(K); IncFII
H102-2	* Klebsiella pneumoniae *	15	IncFIB(K); IncFII(K)
A116-2	* Klebsiella pneumoniae *	2728	IncFIB(K); IncFII(K)
A117-2	* Klebsiella pneumoniae *	16	Col440II; IncFIB(K); IncFII(K)
A118-2	* Klebsiella pneumoniae *	16	Col440II; IncFIB(K); IncFII
A120-1	* Klebsiella pneumoniae *	101	Col440I; IncFIB(K); IncFI(A)
A121-2	* Klebsiella pneumoniae *	17	IncFIB(K); IncR
A127-1	* Klebsiella pneumoniae *	3248	IncFIB(K); IncFII(K)
A135-2	* Klebsiella pneumoniae *	2728	IncFIB(K); IncFII(K)
A139-1	* Klebsiella pneumoniae *	2728	IncFIB(K); IncFII(K)
A143-2	* Klebsiella quasipneumoniae *	1504	IncFIB(K); IncHI1B
A145-2	* Klebsiella pneumoniae *	530	IncFIA(HI1); IncFIB(K); Inc R; IncFII(K)

### Genetic comparison of *Klebsiella* spp. and *E. coli* isolates

Whole genome-based cgMLST phylogenetic analysis of *Klebsiella* spp. isolates was performed and a MST was calculated (Fig. 4). Distance calculation between the 16 samples revealed a maximum allelic distance between samples of 2867 across the MST. Based on the defined complex threshold (CT) of 15 allelic differences, two different clusters were obtained containing five isolates from clinical wounds. The isolates were closely related with a maximum allelic difference of four. Cluster 1 had three ST2728 isolates with similar plasmid incompatibility group IncFIB(K), IncFII(K). Cluster 2 had two ST16 isolates with similar plasmid incompatibility group Col440II, IncFIB(K), IncFII(K). Phylogenetic analysis of *E. coli* isolates was also performed and an MST was calculated based on cgMLST (Fig. 3). Distance calculation between the 16 samples revealed a maximum allelic distance between samples of 2347 across the MST. Based on the defined CT of 10 allelic differences, three different clusters were obtained. The isolates in cluster 1 (Fig. 3) included four isolates from urine of healthy humans and two isolates from healthy poultry animals. The isolates (ST155 and a common plasmid incompatibility group p0111, IncQ1, IncFII) were closely related with a maximum allelic difference of three. The farms where the poultry samples were obtained are in the same location as the healthy individuals. Cluster 2 consists of an ST1722 isolate from a healthy individual (nasal sample) and two ST1722 isolates from clinical wounds that were closely related with an allelic difference of one. The isolates had a common plasmid incompatibility group IncFIA, IncFIB. The location where the isolates from clinical wounds were obtained is not in close proximity to the location where nasal samples of the healthy individuals were collected. Cluster 3 includes two ST10 isolates with similar plasmid incompatibility group IncFII from clinical wounds.

## Discussion

AMR is of global health concern and is increasingly frustrating therapeutic efforts against infectious diseases. This has been linked to the misuse and/or overuse of antimicrobials in humans and animals [9, 11, 29]. The present study determined the frequency of antimicrobial-resistant *E. coli* and *Klebsiella* spp. isolates recovered from humans and poultry in selected areas of Edo state, Nigeria, and provides the genetic characterization of the isolates. Humans and the poultry environment have been previously reported to be reservoirs for antimicrobial-resistant bacteria [9, 11, 29–31]. The frequency of resistant isolates from humans was higher compared to isolates from poultry. Results from this study show that *Klebsiella* spp. and *E. coli* isolates especially from poultry were resistant to antibiotics commonly used in human and veterinary medicine. Although bans on the non-therapeutic use of antimicrobials exist in multiple countries including Nigeria, the coordinated surveillance and monitoring of the use of antimicrobials and their resistance in Nigeria is still limited, which has resulted in an increase of antimicrobial-resistant pathogens. One limitation of this study was the failure to document the use of specific antimicrobials on the farms where samples were collected. Previous studies from developed countries have demonstrated the clonal spread of bacteria between human and animal reservoirs while only a few studies are available from sub-Saharan Africa [30]. Due to limited discriminatory power, classical MLST is not adequate to reaveal the clonal spread of bacteria between human and animal reservoirs. Results from this study suggest clonal transmission of *E. coli* between the two reservoirs (healthy humans and poultry). This correlates with a previous publication that reported potential clonal transmission between the two reservoirs (humans and poultry) in a rural Ghanaian town [30]. Another recent African study, which also correlates with results from the present study, showed a complex relationship between multi-resistant *E. coli* isolates in poultry and humans indicating that multi-resistance control must involve a One-Health approach and a multi-sectoral collaboration [32]. In our study, the isolation of closely related *E. coli* ST1722 isolates from humans that were not in close proximity to each other furthermore indicates clonal spread of resistant bacteria in the human community. The lower level of hygiene in developing countries compared to developed countries facilitates inter-host transmission of bacteria. Previous reports have indicated that contaminated poultry products are a significant source for acquisition of resistant bacteria due to hand contamination and cross-contamination during meal preparation [8, 33, 34].

In our study, no ESBL-producing *E. coli* could be isolated from poultry. This contrasts with previous reports of ESBL-producing *E. coli* detected from poultry [30, 35, 36]. In our study, *bla*_CTX-M-15_ was predominantly present in the resistant isolates obtained from humans. This correlates with a previous study from Nigeria revealing that *bla*_CTX-M-15_ constitutes the most frequent ESBL type detected in clinical human *Enterobacteriaceae* isolates [9]. In our study, the frequency of detection of ESBL *bla*_CTX-M-15_ in bacteria from faecal poultry samples was considerably lower than in human isolates.

*E. coli* ST155, which had a high frequency in human and poultry isolates, has been previously reported as responsible for transmission of ESBL genes and plasmid-mediated spread of antibiotic resistance from animals to humans [37, 38]. It has acquired adaptive traits that increase pathogenicity, colonization and spread in different kinds of niches [39, 40]. A recent Nigerian study reported *E. coli* ST155 as one of the most common STs detected among ESBL *E coli* strains from human, chickens, and chicken market environments [41]. The findings demonstrated that the co-colonization of antimicrobial-resistant *E. coli* from a shared source is also possible, which correlates with results obtained from this study. *K. pneumoniae* ST307 is an important clone that has been identified in diverse locations worldwide and is associated with the ESBL gene *bla*_CTX-M-15_ [42, 43]. *K. pneumoniae* ST307 has been involved in local hospital outbreaks in Africa, the Americas, Asia and Europe [44] with limited reports on non-human sources in Africa [45]. The detection of *K. pneumoniae* ST307 harbouring *bla*_CTX-M-15_ in poultry is of public health importance as it confirms that not only humans could serve as reservoir for this clone. To the best of our knowledge, our results present the first identification of *K. pneumoniae* ST307 *bla*_CTX-M-15_ in poultry in Nigeria. Virulence genes were detected in all the *Klebsiella* spp. and *E. coli* isolates, which suggests poultry may be a reservoir for genes encoding virulence in addition to human sources. *K. pneumoniae* has four important virulence factors, adhesive fimbriae (including type 1 and type 3 fimbriae), capsule, LPS and siderophores, that contribute to the pathogenicity of *K. pneumoniae* isolates. Siderophores were the most significant virulence genes detected among the *Klebsiella* spp. isolates. A previous report [46] showed that acquiring the virulence factor yersiniabactin is usually the initial step in accumulating more potent siderophores to make isolates more invasive. Yersiniabactin genes were found among eight human *K. pneumoniae* isolates and and one poultry isolate. All isolates with yersiniabactin genes were resistant/multidrug-resistant and ESBL-positive, making their infections persistent and antibiotic-resistant and possibly contributing to their dominance over the other pathogroups. The *ent*B gene is a siderophore-associated gene of *K. pneumonia* e. Iron is required for bacterial survival. *K. pneumoniae* usually acquires iron via the secretion of siderophores, which have a higher affinity for iron than host transport proteins [47]. In this study, the *ent*B gene was detected in 100 % of the human and poultry *Klebsiella* spp. isolates which agrees with other reports [47, 48]. *Fim*H and *mrk*D are other important virulent factors detected among the *Klebsiella* spp. isolates essential in pili formation which allows for adherence of the pathogen and for formation of biofilms on biotic and abiotic surfaces. In this present study, the rates of detection of the *fimH* and *mrkD* genes among the human and poultry *Klebsiella* spp. isolates were 100 and 81.3 %, respectively, which is consistent with the results obtained in previous studies [47–49]. The presence of varying virulence markers in the *E. coli* isolates suggests different pathogroups present among the *E. coli* isolates. A previous classification of Uropathogenic *E. coli* (UPEC) considers the presence of at least two or more of the following genes: *chu*A, *fyu*A (coding for ferric yersinia uptake yersiniabactin receptor), *vat* and *yfc*V (adhesin) [50]. Significantly, four *E. coli* isolates in which three were ESBL ST1722 with predicted serotype O1:H25 had the virulence factor *fyu*A detected in association with *yfc*V and *chu*A, confirming them as possible UPEC pathotypes. Previously, Johnson *et al*. [51] classified bacterial strains as possible Extraintestinal pathogenic *E. coli* (ExPEC) isolates when possessing two or more of the following virulence determinants: *pap*AH, and/or *pap*C (P fimbriae), *sfa-foc*DE (S and F1C fimbriae), *afa-dra*BC (Dr- binding adhesins), *iut*A (aerobacting siderophore system) and *kps*M II (group 2 capsules). None of these genes were in the same *E. coli* isolate in this study. However, some genes, such as *pap*C or *kps*M II, in association with other virulence markers were recovered in the study. These isolates could be considered commensals, coding for ExPEC-associated virulence genes. Another frequent virulence marker detected among the *E. coli* isolates is *lpf*A. Previous studies detected *lpf*A in enteropathogenic *E. coli* (EPEC), cattle shiga toxin-producing *E. coli* (STEC), extra-intestinal pathogenic *E. coli* and commensal *E. coli* [52, 53]. This gene was detected in human as well as poultry *E. coli* isolates in this study. In the present study, 100 % of the human and poultry *E. coli* isolates possessed multi-virulence-associated genes. These results agreed with previous studies that reported the majority of *E. coli* isolates to have at least three virulence-associated genes [54, 55]. The results of this study indicated that the examined *E. coli* and *Klebsiella* spp. strains in both human and poultry isolates possess a characteristic set of virulence factors and some of these virulent factors associated with AMR genes may be of public health concern as a result of their potential to cause human infections [29, 56]. Using the MobileElementFinder web tool, a variety in the number and combination of MGEs was identified simplifying the detection and characterization of MGEs and their relationship to AMR and virulence genes for this study. ISs and other transposable elements have previously been reported to be associated with the mobilization of antibiotic-resistant determinants and the transmission of pathogenic characteristics [57]. The detection of MGEs in most of the resistant isolates associated with antimicrobial-resistant and virulence genes is of public health concern as they might possibly facilitate the transmission of these genes among the isolates. A good understanding of the genetic diversity of an organism is necessary to understand its transmission dynamics and to predict its source and potential for transmission from animals to humans [58].The importance of WGS as a vital genomic surveillance tool to detect and characterize the genetic basis of Nigerian human and poultry *Klebsiella* spp. and *E. coli* isolates has been demonstrated in this study

This study describes the detection of ESBL-producing/antimicrobial-resistant bacteria with varying virulence profiles in humans and also poultry samples from Edo state in Nigeria. The results show that poultry farms or meat products might be an important source for ESBL-producing/antimicrobial-resistant pathogenic bacteria. The importance of genomic approaches in the surveillance of AMR for improving the prevention and control of infection in Nigerian hospitals cannot be underestimated. The benefit of public health genomic surveillance of pathogens and its potential for outbreak detection is of great importance in low- and middle-income countries. An integrated ‘One Health’ surveillance system involving collaboration among scientists, health institutions and public health authorities is required to monitor transmission of AMR in Nigeria.

## Supplementary Data

Supplementary material 1
